# 6R-TaS_2_ Anchored on Mo Foil as a Robust Electrocatalyst for Hydrogen Evolution

**DOI:** 10.1021/acsami.5c22479

**Published:** 2025-12-15

**Authors:** Antonia Kagkoura, Filipa M. Oliveira, Kseniia Mosina, Jan Luxa, Zdeněk Sofer

**Affiliations:** Department of Inorganic Chemistry, 52735 University of Chemistry and Technology Prague, Technická 5, Prague 166 28 Prague 6, Czech Republic

**Keywords:** tantalum disulfide, molybdenum, hydrogen evolution reaction, transition metal dichalcogenides, metallic substrates, electrochemical exfoliation, electrocatalysis

## Abstract

Affordable and durable HER catalysts are critical for sustainable hydrogen production. We report electrochemically exfoliated multilayer TaS_2_ flakes, predominantly in the metallic 6R phase, anchored on conductive Mo foil. The optimized hybrid (0.8 mg TaS_2_) initiates HER at −0.06 V vs RHE and reaches −10 mA cm^–2^ at only −150 mV vs RHE, approaching the performance of 20% Pt/C on Mo. Fast kinetics (Tafel slope of 68 mV dec^–1^), low charge-transfer resistance, and a high electrochemically active surface area (∼5.5 cm^2^) contribute to the superior HER activity, while maintaining ∼58% of the initial current after ∼56 h of continuous operation. Compared to TaS_2_ drop-cast on glassy carbon, the Mo substrate boosts performance by facilitating electron transport and proton adsorption. This work demonstrates substrate engineering combined with metallic transition metal dichalcogenide phases as a scalable route to high-performance, earth-abundant HER catalysts. Beyond performance, this study highlights the largely unexplored potential of the 6R polymorph, whose intrinsic metallicity and distinct stacking offer new opportunities for interface engineering in 2D electrocatalysts.

## Graphical abstract

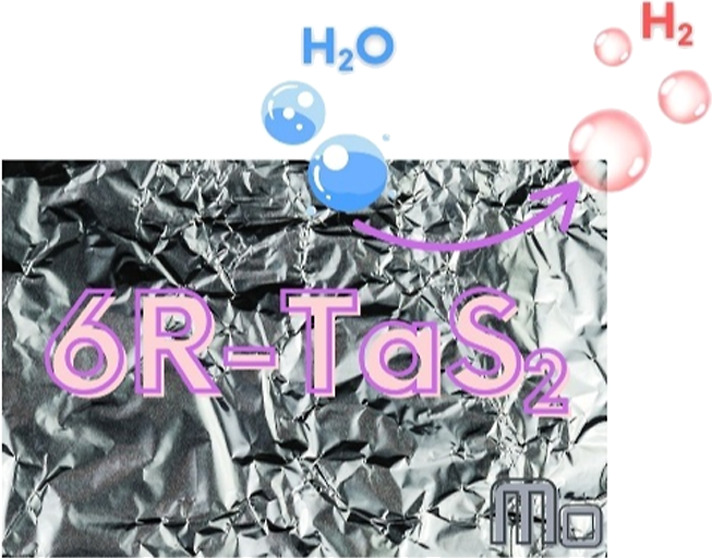

## Introduction

As the hydrogen economy gains momentum as a cornerstone of the shift toward cleaner and more sustainable energy systems, hydrogen (H_2_) stands out as a high-energy fuel (≈120–140 MJ kg^–1^) that generates only water as a byproduct during use.^1,2^ One of the simple ways of making it is electrochemical water splitting, and with renewable energy powering the process, it can assist in mass decarbonization.^1,3^ Central to water splitting is the hydrogen evolution reaction (HER), which entails the reduction of protons or water molecules into hydrogen gas.^4^ Platinum is still the best known HER catalyst, but its high cost and low availability push research into alternative avenues. The transition-metal dichalcogenides (TMDs) have emerged as one of the most promising material families in this direction.^5,24^ Recent studies have demonstrated that 2D materials, including TMDs, can achieve high current density HER, highlighting their potential for practical applications and scalable hydrogen production.^6,7^

Tantalum disulfide (TaS_2_) has recently gained attention as a catalyst for the HER owing to its metallic conductivity—observed across its main polymorphs (1T, 3R, and 6R)—and its good structural stability.^8,9^ Exfoliated TaS_2_ nanosheets provide abundant edge and basal sites, and their surface properties can be further tuned through thermal or electrochemical treatments to optimize hydrogen adsorption.^21^ In particular, the less studied 6R polymorph, combines alternating 1H and 1T layers in a rhombohedral stacking, remains metallic over a wide temperature range and exhibits charge-density-wave transitions at approximately 320 and 305 K.^10,37^ These electronic features make 6R-TaS_2_ especially promising for electrocatalytic applications.

TaS_2_ can be synthesized via methods such as chemical vapor transport (CVT) or direct sulfurization, whereas exfoliation techniques—including liquid-phase exfoliation (LPE), mechanical exfoliation, and electrochemical exfoliation—are commonly employed to obtain few-layer flakes.^11,12^ Compared to mechanical exfoliation, which produces high-quality flakes but at very low yield, and LPE, which often requires toxic solvents, electrochemical exfoliation stands out by balancing scalability, safety, and structural preservation.^12,13^ Although more commonly applied to group 6 TMDs, recent studies have also highlighted its potential for TaS_2_, provided that electrolyte composition and conditions are properly optimized.^13^

Recent progress in exfoliation techniques and the design of non-noble, cost-efficient HER electrocatalysts has significantly expanded the scope of TMD-based materials.^14–16^ For example, liquid-phase and electrochemical exfoliation strategies have been successfully applied to various metallic and semiconducting TMDs, enabling the production of few-layer nanosheets with abundant active sites while maintaining structural integrity.^17–19^ These advances highlight the potential of exfoliated TMD nanostructures as promising platforms for HER electrocatalysis; however, despite their advantages, important challenges remain in closing the performance gap with noble-metal benchmarks.

Although exfoliated TaS_2_ has emerged as a promising HER catalyst,^20–22^ its intrinsic advantages are still insufficient to bridge the performance gap with noble-metal benchmarks. More broadly, TMDs — including TaS_2_ — continue to underperform relative to platinum-group catalysts, primarily due to limitations such as inert basal planes, low densities of active sites, and gradual degradation under prolonged operation.^23^ One practical approach to address the conductivity and stability issues is to combine or integrate them with conductive metallic substrates/platforms.^24,25^ In addition to serving as efficient current collectors, metallic substrates can also promote interfacial charge redistribution, which may expose or activate more catalytic sites at the interface of TMDs.^24,25^

Mo foil stands out among conductive substrates for electrocatalysis due to its high conductivity, chemical stability, and structural compatibility with chalcogenides.^26,27^ These features make it an excellent platform for probing interfacial effects in HER. Previous reports on MoS_2_/Mo systems have demonstrated that using Mo foil not only reduces contact resistance through intimate interfacial contact but, in some cases, also serves as a Mo source during synthesis, enabling seamless integration of active phases such as MoS_2_ or MoO_2_.^28^ This dual role results in electrodes with improved conductivity, enhanced exposure of active sites, and facilitated mass transport. Furthermore, recent studies have highlighted advancements in TMD-based hybrids, including Ni–Co–Mo and MoO_3_ composites, which achieve enhanced HER performance through improved conductivity, increased site density, and optimized electronic structure.^29–32^ These studies highlight the potential of integrating TMDs with conductive supports or cocatalysts to address their intrinsic limitations. This motivates the exploration of TaS_2_/Mo hybrid systems and the systematic investigation of how TaS_2_ loading and interfacial interactions influence the electrocatalytic behavior of metallic TMDs.

However, despite the growing interest in TaS_2_ as a metallic TMD catalyst, systematic studies of TaS_2_/Mo hybrids—particularly those prepared via scalable electrochemical exfoliation followed by postannealing—remain limited. As a result, the interfacial charge-transfer effects and the potential performance advantages of combining TaS_2_ with Mo have not yet been fully elucidated.

In this work, we fabricate and optimize TaS_2_/Mo hybrids by controlling the TaS_2_ loading and evaluate their HER performance in acidic media. We correlate activity gains with charge-transfer kinetics, active surface area, and interfacial effects revealed by Tafel and Electrochemical impedance spectroscopy (EIS) analyses. This study highlights substrate engineering as an easy yet efficient avenue toward enhancing the intrinsic activity of metallic TMD catalysts and a protocol for its extension to other 2D/metal systems for the sustainable evolution of hydrogen.

## Experimental Section

### General

Sulfur (99.999%, <6 mm) and tantalum (99.9%, <100 μm) were purchased from Strem (USA). Molybdenum (Mo) foil with a thickness of 0.2 mm and a purity of >99.9% was purchased from Alfa Aesar (Germany). All other chemicals, reagents, and solvents were purchased from Sigma-Aldrich and used without further purification.

### Synthesis of 6R-TaS_2_ Crystals

6R-TaS_2_ crystals were synthesized via direct reaction of elemental tantalum and sulfur, adapting procedures previously reported for 2H-TaS_2_.^33,34^ Specifically, Ta powder (10 g) and sulfur were combined in a 1:2 stoichiometric ratio, sealed in a quartz ampule (20 × 120 mm) under high vacuum (≈1 × 10^–3^ Pa) using an O_2_/H_2_ torch. The mixture was first heated to 450 °C for 12 h, followed by stepwise heating to 600 °C (48 h) and 900 °C (48 h), and finally cooled to room temperature over 24 h at a controlled rate (±5 °C min^–1^).

### Preparation of Exfoliated TaS_2_

TaS_2_ was obtained via electrochemical exfoliation in a 0.05 M solution of tetrabutylammonium fluoride (TBAF) in acetonitrile, applying a voltage range from 0 to −6 V for 100 s.

### Preparation of TaS_2_/Mo

TaS_2_ (0.8 mg) was dispersed in methanol and drop-cast onto a 2 × 2 cm^2^ Mo substrate placed on a hot plate at 40 °C to facilitate solvent evaporation. The resulting films were then annealed at 600 °C for 2 h under a 95% Ar/5% H_2_ atmosphere to improve adhesion and crystallinity. Finally, the films were cut into 0.5 × 0.5 cm^2^ pieces for further characterization. **0.4** and **1.5 TaS**_**2**_ **/Mo** were prepared as described above with initial concentrations of 0.4 and 1.5 mg TaS_2_, respectively.

### Pt/C/Mo

0.8 mg of 20 wt % Pt on graphitized carbon (Pt/C) were dispersed in methanol and drop-cast onto a 2 × 2 cm^2^ Mo substrate, which was placed on a hot plate at 40 °C to facilitate solvent evaporation.

### Microscopy Techniques

The morphology of the studied materials was examined using a Tescan MAIA-3 Field Emission Scanning Electron Microscope (FEG-SEM). Energy-dispersive X-ray spectroscopy (EDS) was carried out to determine elemental composition and perform elemental mapping, using an 80 mm^2^ SDD detector (Oxford Instruments) and the AZtecEnergy software suite. For SEM/EDS analysis, samples were mounted on carbon conductive tape. Transmission electron microscopy (TEM) was conducted on a Jeol 2200 FS EFTEM microscope (Jeol, Japan) operated at an acceleration voltage of 200 kV. Images were captured with an SIS MegaView III digital camera (Soft Imaging Systems) and processed using AnalySIS v. 2.0 software. Elemental mapping via TEM employed an X-MaxN 80 TS SDD detector (Oxford Instruments, UK). For TEM sample preparation, materials were dispersed in pure ethanol, drop-cast onto Cu TEM grids (200 mesh, Formvar/carbon support, TED PELLA, Inc.), and dried overnight at ambient temperature.

### X-Ray Diffraction (XRD)

Powder X-ray diffraction patterns were recorded at room temperature using a Bruker D8 Discover diffractometer (Bruker, Germany) configured in a parafocusing Bragg–Brentano geometry, employing Cu Kα radiation (λ = 0.15418 nm, 40 kV, 40 mA). The scans were performed over the 2θ range of 5°–70°, with a step size of 0.020°. Data analysis was carried out using the EVA software package.

### X-Ray Photoelectron Spectroscopy (XPS)

XPS was carried out on a Phoibos 100 (Specs, Germany) with monochromatic Al source (Kα1 = 1486.7 eV). For the measurements, the samples were attached onto a Cu conductive tape. High-resolution core-level spectra were recorded with an *E*_pass_ = 40 eV and a step of 0.1 eV. Compensation using a flood gun was used to a yield C 1s peak position at 284.8 eV.

### Raman Spectroscopy

Raman spectra were acquired using a Renishaw inVia Raman microscope (Renishaw, UK) in backscattering geometry with a CCD detector. A 532 nm DPSS laser (50 mW) was used, with the measurement conducted at 5 mW laser power and using a 50× objective lens. Instrument calibration was performed with a silicon reference sample, yielding a peak at 520 cm^–1^ and a spectral resolution better than 1 cm^–1^. For sample preparation, materials were suspended in deionized water (1 mg/mL), ultrasonicated for 10 min, drop-cast onto a silicon wafer, and dried prior to analysis.

### Atomic Force Microscopy (AFM)

AFM images were collected via a Nanosurf FlexAFM in ambient conditions. Semicontact AC (tapping) mode was utilized for data acquisition with a set point of around ∼62%. Tap 190 Al-G silicon tips with a tip radius of 7 nm and a resonant frequency of 190 kHz were used. Images were processed with Gwyddion software.

### Electrochemical Characterization for the Hydrogen Evolution Reaction (HER)

Electrochemical measurements were carried out using linear sweep voltammetry (LSV) with an Autolab PGSTAT 204 potentiostat (Metrohm, Switzerland). For glassy carbon (GC) measurement, a conventional three-electrode cell was employed, featuring a rotating disk electrode (RDE) with a GC disk (geometric area: 0.196 cm^2^) as the working electrode, a graphite rod as the counter electrode, and a Hg/HgSO_4_ reference electrode (in 0.5 M K_2_SO_4_). The HER activity was assessed in Ar-saturated 0.5 M H_2_SO_4_ electrolyte at room temperature. LSV data were corrected for *iR* drop by applying a 5–10% compensation, depending on the specific sample resistance, to account for solution ohmic losses. The catalyst ink was prepared by dispersing 4.0 mg of catalytic powder in 1 mL of a solvent mixture containing deionized water, isopropanol, and 5% Nafion solution in a 4:1:0.02 volume ratio. The mixture was sonicated for 30 min to ensure uniform dispersion. Prior to catalyst deposition, the working electrode was polished using alumina slurry, rinsed with deionized water, and cleaned via sonication in double-distilled water. Subsequently, 8.5 μL of the ink was drop-cast onto the electrode surface.

In the case of the TaS_2_/Mo films, 0.5 × 0.5 cm^2^ pieces were evaluated for the HER. The films were held in place using a commercial electrode holder equipped with a spring-loaded clip to ensure stable electrical contact throughout the electrochemical measurements.

Electrochemical impedance spectroscopy (EIS) measurements were conducted in the frequency range of 10^5^–10^–1^ Hz with a 10 mV AC amplitude. EIS data were collected at a potential corresponding to a HER current density of approximately −1.95 mA cm^–2^.

## Results and Discussion

Single-phase 6R-TaS_2_ crystals were obtained by directly reacting elemental tantalum powder with sulfur granules in a sealed quartz ampule. (see Experimental part for more details).^33^ Multilayer TaS_2_ flakes were obtained via electrochemical exfoliation in a 0.05 M tetrabutylammonium fluoride (TBAF) solution in acetonitrile. This method successfully produced predominantly 6R-phase TaS_2_ flakes, a metallic polymorph whose conductivity and catalytic properties are comparable to those of the 1T phase, making it attractive for electrocatalytic applications. Transmission electron microscopy (TEM) reveals the characteristic layered morphology of the exfoliated TaS_2_ flakes. Elemental mapping further shows a uniform and colocalized distribution of Ta and S, confirming the stoichiometric integrity and high crystallinity of the material (Figure 1a). No evidence of impurities or phase segregation is observed in the flakes’ interior, consistent with the single-phase 6R-TaS_2_ structure confirmed by XRD.

**Figure 1:**
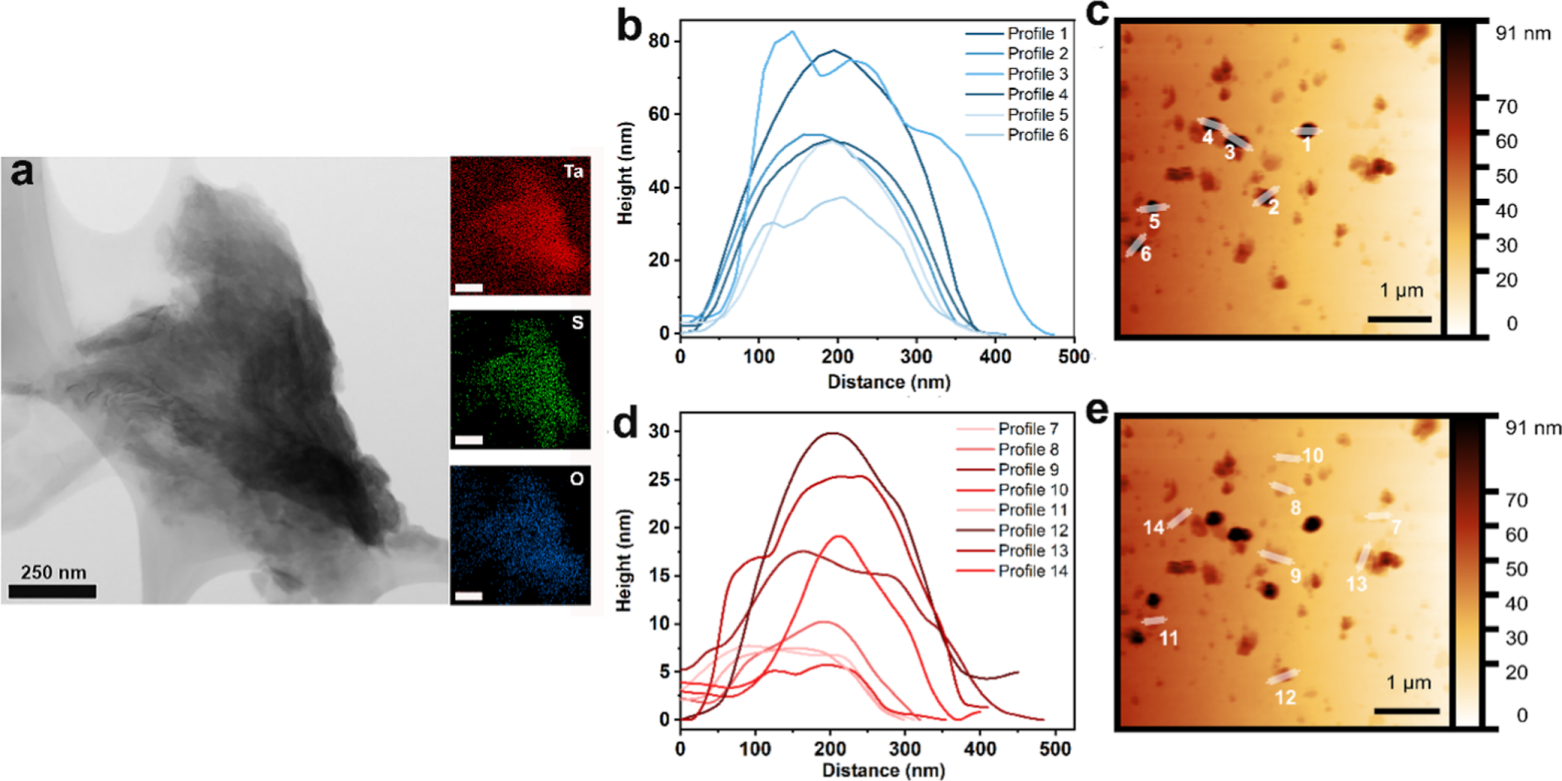
(a) TEM image of exfoliated TaS_2_ with corresponding EDS elemental maps showing the distribution of Ta and S (scale bars for elemental mapping: 250 nm). (b,d) AFM height profiles (distance vs. height) extracted along the indicated lines. (c,e) AFM topographic images of multilayer TaS_2_ flakes with thicknesses ranging from 10 to 80 nm and the corresponding line scan positions marked.

Atomic force microscopy (AFM) was performed to investigate the thickness and lateral dimensions of exfoliated TaS_2_ flakes. AFM analysis revealed multilayer flakes with a broad distribution of thicknesses and lateral dimensions (Figure 1b–e). Flakes with thicknesses up to ∼80 nm were observed, with an average lateral size of ∼400 nm. In particular, the flakes shown in Figure 2b,c exhibit lateral dimensions of ∼400 nm and thicknesses of 50–80 nm, while thinner flakes in Figure 2d,e display lateral sizes of ∼300 nm and thicknesses of 10–30 nm. These results confirm the successful exfoliation of TaS_2_ into multilayer flakes of varying dimensions, consistent with TEM analysis.

**Figure 2:**
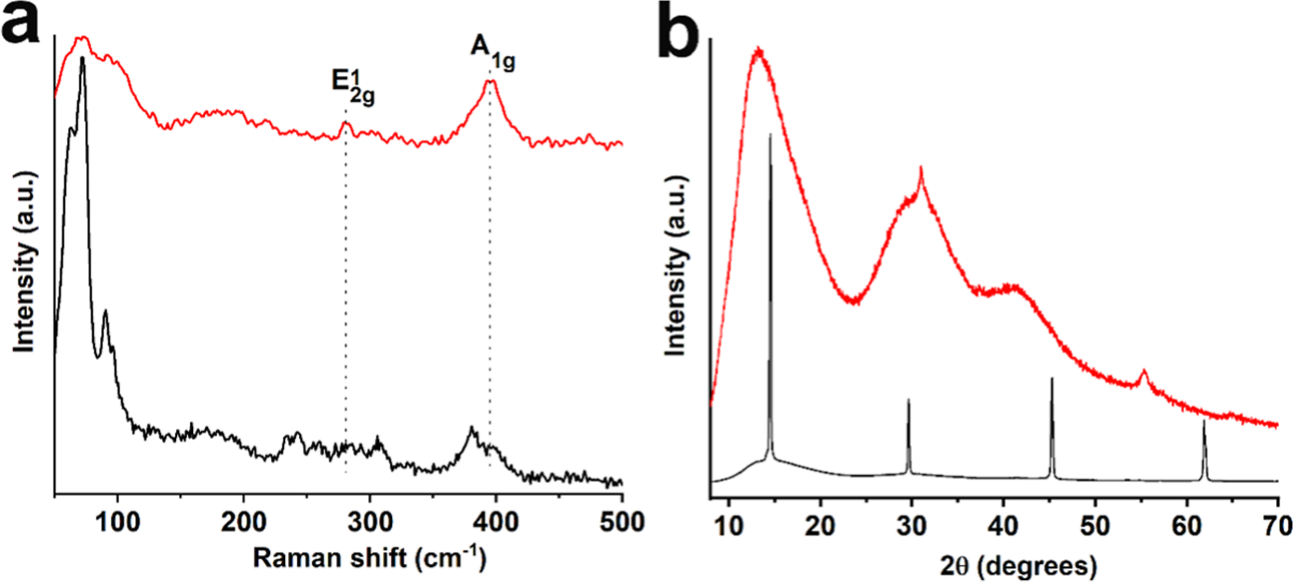
(a) Raman spectra and (b) XRD patterns of exfoliated (red) and bulk (black) TaS_2_.

Raman spectroscopy confirmed the coexistence of the 6R-phase, as the exfoliated TaS_2_ flakes exhibit vibrational features characteristic of the alternating 1H/1T stacking of the 6R polymorph (Figure 2a).^10,37^ Specifically, layers with trigonal prismatic coordination of Ta atoms exhibit characteristic vibrational modes, including the A_1g_ out-of-plane mode at approximately 395 cm^–1^, the E^1^_2g_ in-plane mode near 280 cm^–1^, and a broad second-order peak at approximately 180 cm^–1^, associated with two-phonon processes.^35,36^ In contrast, layers with octahedral Ta coordination present folded-back optical modes at approximately 80, 296, and 380 cm^–1^, attributed to the formation of commensurate domains at room temperature.^36^ The Raman spectrum of 6R-TaS_2_ exhibits a sharp peak at 243 cm^–1^ and well-resolved low-frequency phonon modes at around 70, 80, and 98 cm^–1^.^37^ The spectroscopic features are a result of Brillouin zone folding and provide direct evidence for the presence of a commensurate charge density wave (CCDW) phase in the 1T layers at room temperature.^37^ Bulk TaS_2_ displays analogous Raman bands, consistent with those of the exfoliated material. Additionally, the band observed at 296 cm^–1^ in the exfoliated TaS_2_ appears around 306 cm^–1^ in the bulk material, suggesting that this blue shift likely arises from the reduction of long-range Coulombic interlayer interactions as the number of layers decreases.^35^ Furthermore, the crystalline phase of bulk and exfoliated TaS_2_ was investigated using X-ray diffraction (XRD), as seen in Figure 2b. The XRD patterns of bulk TaS_2_, exhibit characteristic reflections at approximately 14.2°, 29.6°, 45.9°, and 62.6°, corresponding to the planes of the hexagonal 6R phase (ICSD-52117).^35^ Compared to bulk TaS_2_, the XRD pattern of the exfoliated material shows significant broadening of the diffraction peaks, indicating a reduced crystallite size and increased structural disorder.^35^ Notably, the main peak at ∼14.2° in the bulk shifts to ∼13.1° in the exfoliated sample and the higher-angle reflection shifting from ∼45.9° to ∼42.0°. These low-angle shifts indicate an increase in interlayer spacing resulting from the exfoliation process.

Next, exfoliated TaS_2_ was deposited onto Mo foil, which served as a conductive substrate for evaluating its electrocatalytic activity toward the HER. To assess the effect of TaS_2_ loading, three different amounts —0.4, 0.8, and 1.5 mg— were drop-cast onto 2 × 2 cm^2^ Mo foils while placed on a hot plate to promote uniform dispersion. The samples were subsequently annealed at 600 °C for 2 h under Ar/H_2_ atmosphere, leading to 0.4 TaS_2_/Mo, 0.8 TaS_2_/Mo, and 1.5 TaS_2_/Mo materials. After annealing, the Mo foils were cut into 0.5 × 0.5 cm^2^ pieces for electrochemical testing. Scanning electron microscopy (SEM) was performed to examine the surface morphology of TaS_2_-coated Mo substrates with varying loadings (0.4 mg, 0.8 mg, and 1.5 mg), as seen in Figure S1. The SEM images reflected distinct variations in surface coverage and distribution of TaS_2_ flakes as a function of loading, providing information on the quality of dispersion and homogeneity of TaS_2_ layers over Mo support.

X-ray photoelectron spectroscopy (XPS) measurements were performed to characterize the surface of the materials. As shown in Figure 3, the high-resolution core-level spectra of Ta 4f region show four prominent peaks. The spectra are dominated by a doublet at higher binding energies (26.2 and 28.2 eV for Ta 4f_7/2_ and Ta 4f_5/2_, respectively) originating from an oxidized sample surface (Ta_2_O_5_) in all samples. A much less prominent spin–orbit doublet associated with TaS_2_ (23.2 and 25.2 eV for Ta 4f_7/2_ and Ta 4f_5/2_, respectively) was also observed together with O 2s peak (21.6 eV). The exfoliated 6R-TaS_2_ exhibited the lowest degree of oxidation with the TaS_2_/Mo hybrid materials almost completely oxidized to Ta_2_O_5_. These results indicate substantial surface oxidation occurring during the exfoliation process, with further oxidation taking place during the synthesis of the hybrid materials. Moreover, Raman spectra of TaS_2_/Mo samples after annealing at 600 °C confirm that the 6R phase is preserved, indicating that the thermal treatment does not alter the crystal structure (Figure S2).

**Figure 3:**
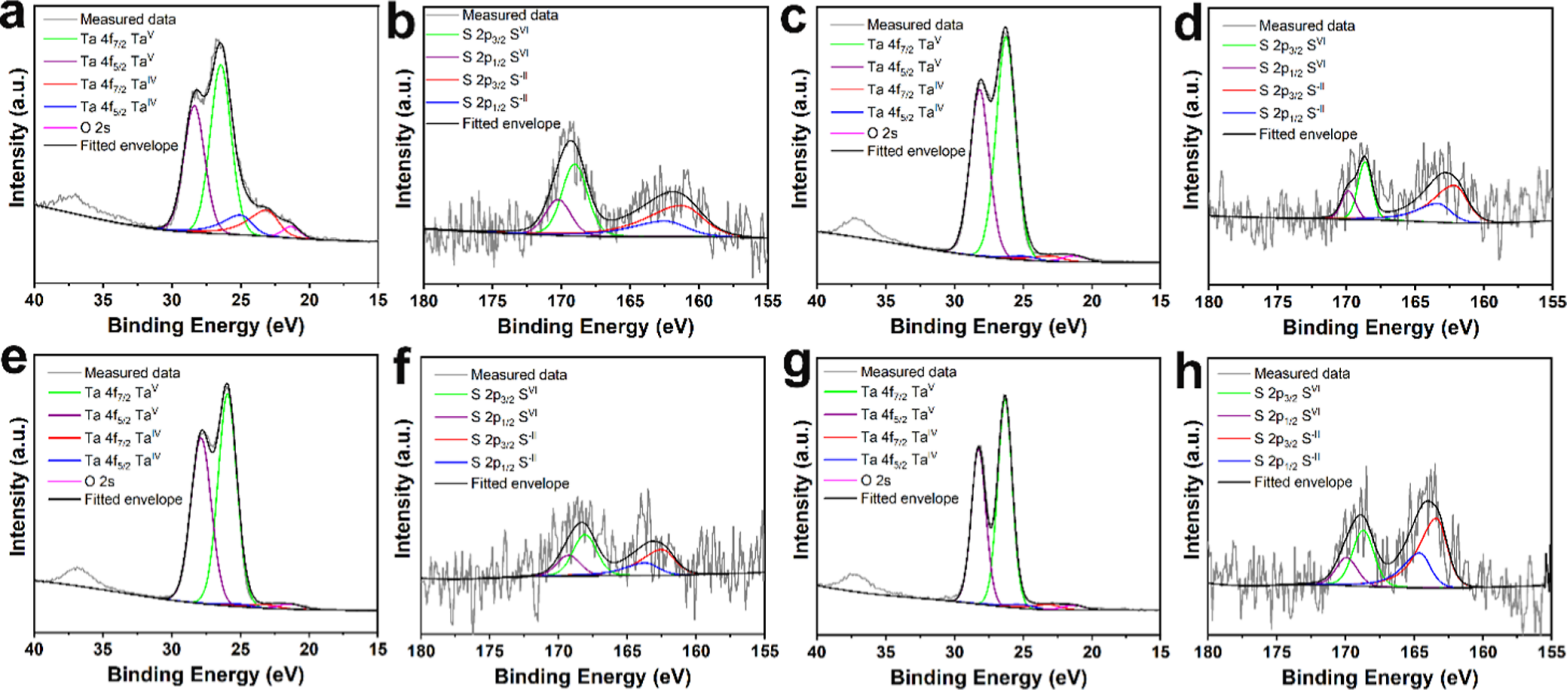
Deconvoluted X-ray photoelectron spectra of (a,b) exfoliated TaS_2_, (c,d) 0.4 TaS_2_/Mo, (e,f) 0.8 TaS_2_/Mo, and (g,h) 1.5 TaS_2_/Mo, showing Ta 4f and S 2p spin–orbit doublets.

The electrocatalytic performance of TaS_2_-modified Mo electrodes, along with reference samples (pristine Mo and annealed Mo), and a benchmark Pt/C catalyst (20 wt % Pt on graphitized carbon deposited on Mo, Pt/C/Mo) (Figure 4), was evaluated for the HER using linear sweep voltammetry (LSV) in 0.5 M H_2_SO_4_ aqueous electrolyte (Figure 4). For the measurements, 0.5 × 0.5 cm^2^ electrode pieces were mounted on a holder and partially immersed in the electrolyte, ensuring that only the active material was in contact with the solution, while the metallic part of the holder remained above the liquid level to avoid interference.

**Figure 4:**
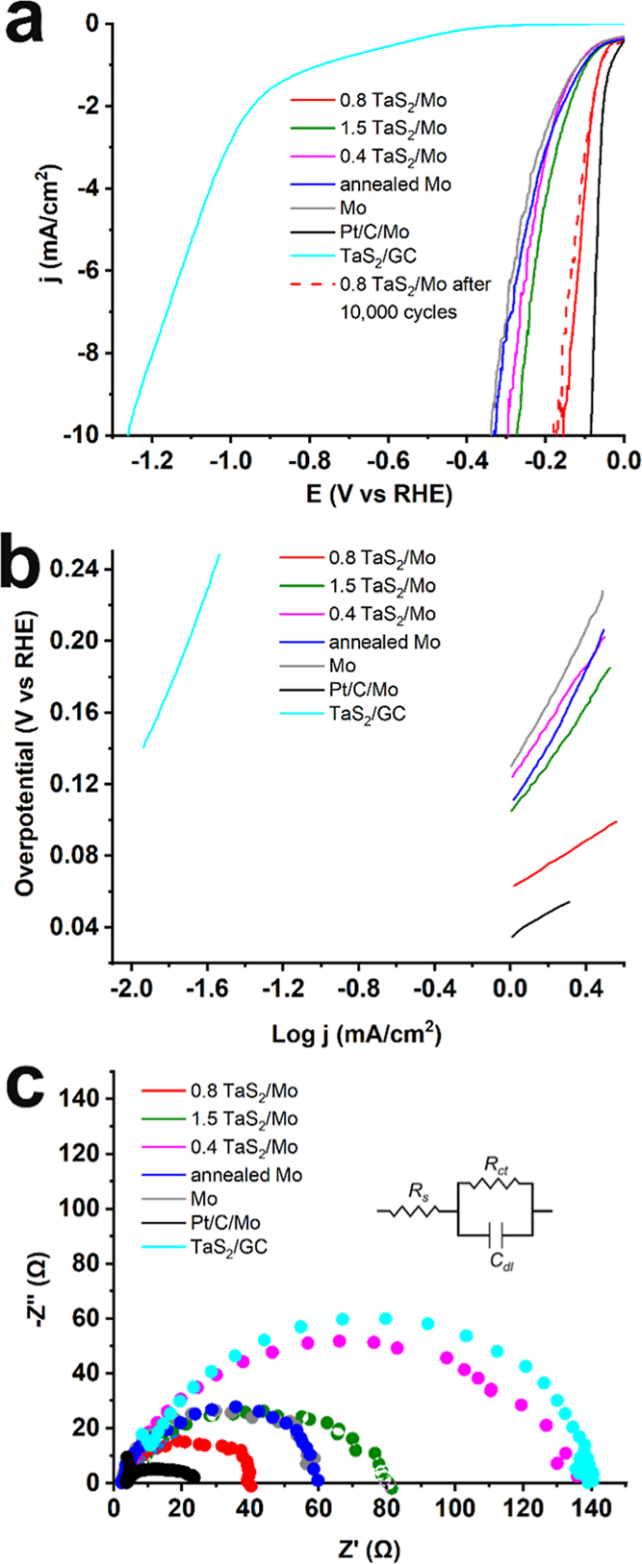
(a) *iR*-corrected LSVs for HER obtained at 5 mV/s scan rate before (solid lines) and after 10,000 cycles (dashed lines) in aqueous 0.5 Μ H_2_SO_4_, (b) Tafel slopes, and (c) Nyquist plots for 0.8 TaS_2_/Mo (red), 1.5 TaS_2_/Mo (green), 0.4 TaS_2_/Mo (pink), annealed Mo (blue), Mo (gray), Pt/C (black) and TaS_2_/GC.

The 0.8 TaS_2_/Mo hybrid demonstrates superior electrocatalytic activity, significantly outperforming compositions with both lower and higher TaS_2_ content (Figure 4a). Hydrogen bubble evolution initiates at −0.06 V vs RHE (−1 mA/cm^2^), which is 40 and 50 mV lower than that of 1.5 TaS_2_/Mo and 0.4 TaS_2_/Mo, respectively, and only 26 mV higher than that of Pt/C. In contrast, annealed Mo and pristine Mo exhibit higher onset potentials of −0.12 V vs RHE. At the benchmark current density of −10 mA/cm^2^, the 0.8 TaS_2_/Mo electrode achieves an overpotential of only 150 mV (−0.15 V vs RHE), which is 120 and 140 mV lower than those of 1.5 TaS_2_/Mo and 0.4 TaS_2_/Mo, respectively. Interestingly, the 0.8 TaS_2_/Mo hybrid exhibits an overpotential only 70 mV higher than that of Pt/C/Mo, highlighting its outstanding HER performance. In contrast, annealed and pristine Mo display considerably higher overpotentials of 330 and 340 mV, respectively.

The reaction mechanism was elucidated through analysis of Tafel slopes derived from the LSV curves and by EIS, as presented in Figure 4b,c, respectively. In agreement with the LSV data, 0.8 TaS_2_/Mo exhibited the lowest Tafel slope among TaS_2_-based materials of 68 mV/dec, indicating that the Heyrovsky step governs the rate-determining process. In this mechanism, protons are initially adsorbed on the electrode surface via the Volmer step, followed by hydrogen evolution through the desorption of adsorbed hydrogen atoms (Heyrovsky step). By comparison, the Pt/C/Mo benchmark shows a Tafel slope of 58 mV/dec, while 1.5 TaS_2_/Mo, 0.4 TaS_2_/Mo, and both annealed and pristine Mo displayed significantly higher Tafel slopes of 148, 160, 206, and 195 mV/dec, respectively, implying slower kinetics limited by proton adsorption.

EIS measurements further corroborate these findings. Performed at a potential corresponding to −1.95 mA/cm^2^ and fitted using a Randles equivalent circuit, the Nyquist plots revealed that 0.8 TaS_2_/Mo possesses the lowest charge transfer resistance (*R*_ct_ = 37.5 Ω), markedly lower than that of 1.5 TaS_2_/Mo (76.2 Ω) and 0.4 TaS_2_/Mo (128 Ω), highlighting its enhanced conductivity. In comparison, pristine and annealed Mo exhibited higher *R*_ct_ values of 54.6 and 57.3 Ω, respectively, consistent with their slower charge-transfer kinetics. The Pt/C/Mo benchmark shows an even lower *R*_ct_ of 22 Ω. EIS parameters (*R*_ct_, Ohmic resistance *R*_s_ and double-layer capacitance *C*_dl_) are incorporated in Table S1.

For comparison, the HER activity of TaS_2_/Mo hybrids was benchmarked against recent reports on Mo-based substrates and TaS_2_-based catalysts (Table S2). TaS_2_/Mo hybrid demonstrates competitive activity compared to state-of-the-art non-noble HER catalysts, with particularly low overpotential and favorable Tafel slope, underscoring the benefits of interfacial engineering.

Moving forward, the stability of the 0.8 TaS_2_Mo electrode was evaluated through 10,000 consecutive electrocatalytic cycles, as shown in Figure 4a. Notably, the TaS_2_-modified Mo with intermediate TaS_2_ content exhibited only a minor potential shift of 30 mV, demonstrating excellent durability under prolonged operation. In addition, the chronoamperometric test conducted at −0.089 V vs RHE for 200,000 s revealed a gradual decrease in current density of ∼42% loss, demonstrating robust durability (Figure 5).

**Figure 5:**
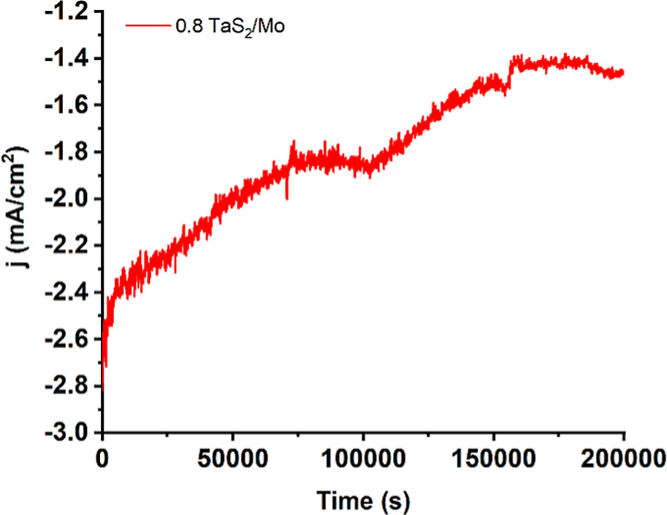
Chronoamperometric response at −0.089 V (versus RHE) for 200,000 s for 0.8 TaS_2_/Mo.

The electrochemically active surface area (ECSA) is a key parameter for evaluating charge transport characteristics in hybrid electrocatalysts. It was estimated using the relation ECSA = *C*_dl_/*C*_s_, where *C*_dl_ is the electrochemical double-layer capacitance and *C*_s_ is the specific capacitance of a smooth surface, assumed as 40 μF/cm^2^. For this calculation, cyclic voltammograms of the TaS_2_-modified Mo and nonmodified Mo materials were acquired within a non-Faradaic potential window at scan rates ranging from 50 to 500 mV/s^1^ (Figure S3). The 0.8 TaS_2_/Mo electrode displayed the highest ECSA value of approximately 5.5 cm^2^, whereas the other TaS_2_-modified and bare Mo samples displayed lower values ranging from 1.77 to 2.48 cm^2^. After 10,000 cycles, the best TaS_2_/Mo composition retained a relatively high ECSA value of 4.0 cm^2^, indicating only a moderate decrease in the electrochemically active area. These results are consistent with the general electrocatalytic activity: the 0.8 TaS_2_/Mo hybrid provides a high active site density and an enlarged surface area due to the optimized TaS_2_ content, leading to enhanced interfacial contact with the Mo substrate and facilitating efficient charge transport. High ECSA values are directly associated with a greater density of catalytic sites, which in turn leads to enhanced HER activity for this composition. To assess intrinsic activity, the current densities were normalized to the electrochemically active surface area (*j*_ECSA_ = (*j*_geo_·*A*_geo_)/ECSA, *A*_geo_ = 0.25 cm^2^, *j*_geo_ at −10 mA·cm^–2^). This analysis reveals that, although the 0.8 TaS_2_/Mo hybrid exhibits the lowest overpotential, its *j*_ECSA_ is lower than that of the 1.5 TaS_2_/Mo sample, indicating that the enhanced geometric performance primarily arises from a favorable structural balance with abundant accessible sites, rather than from intrinsically higher activity per site. A low TaS_2_ content (0.4 TaS_2_/Mo) provides insufficient active sites, while a higher content (1.5 TaS_2_Mo) promotes agglomeration and partial restacking, as also visible in SEM images (Figure S1), which hinders site accessibility and interfacial electron transfer. Analysis of ECSA, *C*_dl_, and *j*_ECSA_ quantitatively supports this observation, and the differences in *R*_ct_ between 0.4 and 1.5 TaS_2_ are consistent with the interplay between active site density and charge-transfer efficiency. Finally, the metallic 6R phase of TaS_2_, present in all hybrids, facilitates charge mobility and proton adsorption, thereby supporting efficient interfacial electron transfer and contributing to the overall HER performance. The presence of surface Ta oxide may also play a supplementary role in modulating the catalytic activity.

To further emphasize the importance of the Mo substrate in the hybrid architecture, exfoliated TaS_2_ was also evaluated as a drop-cast catalyst on a glassy carbon (GC) electrode (TaS_2_/GC). TaS_2_ on GC exhibits a relatively high onset potential of −0.89 V and an overpotential of −1.26 V at a current density of −10 mA/cm^2^, indicating sluggish catalytic activity (Figure 4a). This poor performance is further corroborated by a large Tafel slope of 252 mV/dec (Figure 4b) and a high charge transfer resistance of 129.1 Ω (Figure 4c), reflecting slow reaction kinetics and inefficient electron transfer at the electrode interface.

For a fair comparison between the 0.8 TaS_2_/Mo hybrid and exfoliated TaS_2_ deposited on the GC, the polarization curves were expressed as mass-normalized currents (mA/mg), as seen in Figure S4. The commonly adopted geometric benchmark of −10 mA/cm^2^ was converted to its mass-normalized equivalent, taking into account the actual catalyst loading and electrode area (50 mA/mg for TaS_2_/Mo and 57.6 mA/mg for the GC electrode). The onset potential, conventionally defined at −1 mA/cm^2^ (geometric), is also reported for consistency with HER evaluation standards. However, the performance discussion mainly focuses on the −10 mA/cm^2^ benchmark, where differences in activity are more evident. Under these conditions, the 0.8 TaS_2_/Mo hybrid initiates hydrogen evolution considerably earlier than TaS_2_/GC and requires a lower overpotential of 1.1 V at the same benchmark current, confirming the advantageous role of the conductive Mo substrate. Beyond serving as structural support, Mo facilitates charge transport and proton adsorption, which together enhance the intrinsic activity of the TaS_2_ catalyst.

## Conclusions

This study demonstrates that anchoring electrochemically exfoliated 6R-phase multilayer TaS_2_ flakes onto Mo foil produces a hybrid electrode with superior HER activity compared to either pristine Mo or TaS_2_ drop-cast on glassy carbon. The optimized 0.8 mg TaS_2_/Mo hybrid achieves an onset potential of −0.06 V and requires only 150 mV to reach −10 mA/cm^2^ in acidic media—just 70 mV higher than 20% Pt/C on Mo. It combines fast charge-transfer kinetics (low Tafel slope, minimal *R*_ct_) with a high active surface area (∼5.5 cm^2^, retaining 4.0 cm^2^ after 10,000 cycles). During long-term chronoamperometry over ∼56 h, the electrode retains ∼58% of its initial current, demonstrating reasonable stability under extended operation. The improvement arises not only from the metallic 6R phase of TaS_2_ but also from the Mo substrate, which facilitates electron transport and proton adsorption at the interface. These findings underscore substrate engineering as a practical strategy to enhance 2D chalcogenide catalysts and can be extended to other TMD/metal systems through interface tuning or doping. Moreover, the demonstration of HER activity in the less-explored 6R polymorph opens opportunities for systematic studies on its unique electronic structure and interfacial chemistry, which may further unlock performance gains beyond those of the widely studied 2H phase.

## Supplementary Material



## Data Availability

The data sets generated during and/or analyzed during the study are accessible via the Zenodo repository: https://zenodo.org/records/16737383

## Notes

SEM images of (a) 0.4 TaS_2_/Mo, (b) 0.8 TaS_2_/Mo, (c) 1.5 TaS_2_/Mo, and (d) plain Mo substrate. Raman spectra of 0.4 TaS_2_/Mo (pink), 0.8 TaS_2_/Mo (red), and 1.5 TaS_2_/Mo (green). Cyclic voltammograms of (a) 0.8 TaS_2_/Mo, (b) 0.4 TaS_2_/Mo, (c) 1.5 TaS_2_/Mo, (d) annealed Mo (e) Mo and (f) 0.8 TaS_2_/Mo after 10,000 cycles. Table for electrocatalytic HER parameters in for all tested materials. Comparison table of Mo- and TaS_2_ based electrocatalysts for HER and Mass-normalized polarization curves (current density per catalyst mass, mA/mg) for TaS_2_/GC (black) and 0.8 TaS_2_/Mo electrode (red) (PDF)

## References

[1] Jia Q., Zhang T., Zhu Z., Cai R., Song K., Yan F., Qayyum A. (2025). Harnessing
Hydrogen Energy Storage for Renewable Energy
Stability in China: A Path to Carbon Neutrality. Int. J. Hydrogen Energy.

[2] Boretti A., Pollet B. G. (2024). Hydrogen Economy:
Paving the Path to a Sustainable,
Low-Carbon Future. Int. J. Hydrogen Energy.

[3] Staffell I., Scamman D., Velazquez
Abad A., Balcombe P., Dodds P. E., Ekins P., Shah N., Ward K. R. (2019). The Role of Hydrogen
and Fuel Cells in the Global Energy System. Energy Environ. Sci..

[4] Ock I. W., Yin J., Wang S., Zhao X., Baik J. M., Chen J. (2025). Advances in
Blue Energy Fuels: Harvesting Energy from Ocean for Self-Powered Electrolysis. Adv. Energy Mater..

[5] Sim Y., Chae Y., Kwon S.-Y. (2022). Recent Advances in Metallic Transition
Metal Dichalcogenides as Electrocatalysts for Hydrogen Evolution Reaction. iScience.

[6] Luo Y., Tang L., Khan U., Yu Q., Cheng H.-M., Zou X., Liu B. (2019). Morphology and Surface
Chemistry Engineering toward
PH-Universal Catalysts for Hydrogen Evolution at High Current Density. Nat. Commun..

[7] Liu H., Xie R., Luo Y., Cui Z., Yu Q., Gao Z., Zhang Z., Yang F., Kang X., Ge S., Li S., Gao X., Chai G., Liu L., Liu B. (2022). Dual Interfacial
Engineering of a Chevrel Phase Electrode Material for Stable Hydrogen
Evolution at 2500 MA Cm^–2^. Nat. Commun..

[8] Yu Q., Zhang Z., Qiu S., Luo Y., Liu Z., Yang F., Liu H., Ge S., Zou X., Ding B., Ren W., Cheng H.-M., Sun C., Liu B. (2021). A Ta-TaS_2_ Monolith Catalyst with Robust and Metallic Interface
for Superior Hydrogen Evolution. Nat. Commun..

[9] Najafi L., Bellani S., Oropesa-Nuñez R., Martín-García B., Prato M., Pasquale L., Panda J.-K., Marvan P., Sofer Z., Bonaccorso F. (2020). TaS_2_, TaSe_2_, and Their Heterogeneous Films as Catalysts for
the Hydrogen Evolution
Reaction. ACS Catal..

[10] Pal S., Bahera P., Sahu S. R., Srivastava H., Srivastava A. K., Lalla N. P., Sankar R., Banerjee A., Roy S. B. (2023). Charge Density Wave and Superconductivity in 6R-TaS_2_. Physica B Condens. Matter.

[11] Zhao B., Shen D., Zhang Z., Lu P., Hossain M., Li J., Li B., Duan X. (2021). 2D Metallic
Transition-Metal Dichalcogenides:
Structures, Synthesis, Properties, and Applications. Adv. Funct. Mater..

[12] Zhao M., Casiraghi C., Parvez K. (2024). Electrochemical Exfoliation of 2D
Materials beyond Graphene. Chem. Soc. Rev..

[13] Chen H., Si J., Lyu S., Zhang T., Li Z., Lei C., Lei L., Yuan C., Yang B., Gao L., Hou Y. (2020). Highly Effective
Electrochemical Exfoliation of Ultrathin Tantalum Disulfide Nanosheets
for Energy-Efficient Hydrogen Evolution Electrocatalysis. ACS Appl. Mater. Interfaces.

[14] Li J., Yang X., Zhang Z., Yang W., Duan X., Duan X. (2024). Towards the scalable synthesis of two-dimensional heterostructures
and superlattices beyond exfoliation and restacking. Nat. Mater..

[15] Tian W., Kang M.-A., Shakya J., Li Q., Sui X., Liu M., Wang H., Hamedi M. M. (2025). Liquid-Phase
Exfoliation of 2D Transition
Metal Dichalcogenide Nanosheets in Water. Chem.
Eng. J..

[16] Berni A., García-Guzmán J. J., Alcántara R., Palacios-Santander J.
M., Amine A., Cubillana-Aguilera L. (2024). Rapid and
Eco-Friendly Ultrasonic Exfoliation of Transition Metal Dichalcogenides
Supported on Sonogel-Nanocarbon Black: A Non-Precious Electrocatalyst
for Hydrogen Evolution Reaction. Int. J. Hydrogen
Energy.

[17] Zheng W., Li Y., Liu M., Lee L. Y. S. (2021). Few-Layer Tellurium: Cathodic Exfoliation
and Doping for Collaborative Hydrogen Evolution. Small.

[18] Wang Y., Mayorga-Martinez C. C., Chia X., Sofer Z., Mohamad
Latiff N., Pumera M. (2019). Bipolar Electrochemistry as a Simple
Synthetic Route toward Nanoscale Transition of Mo_2_B_5_ and W_2_B_5_ for Enhanced Hydrogen Evolution
Reaction. ACS Sustain. Chem. Eng..

[19] Jarolímková J., Neubertová V., Severa K., Daniš S., Cais J., Lyutakov O., Kolská Z. (2025). Hydrogen Evolution
Reaction at Different PH of 2D MoS_2_ Prepared via Liquid-Phase
Exfoliation. Surf. Interfac.

[20] Buravets V., Hosek F., Lapcak L., Miliutina E., Sajdl P., Elashnikov R., Švorčík V., Lyutakov O. (2023). Beyond the Platinum Era–Scalable Preparation
and Electrochemical Activation of TaS_2_ Flakes. ACS Appl. Mater. Interfaces.

[21] Yang H., Liu X., He J., Yan J., Bai Y., Yin S., Li H., Yan H. (2025). S-Vacancy-Rich 1T-TaS_2_/Cu_2_S Heterostructures
on Cu Foil for Alkaline Hydrogen Evolution Reaction. ACS Appl. Nano Mater..

[22] Shiraz H. G., Vagin M., Khan Z. U., Chmielowski R., Crispin R., Berggren M. (2025). TaS_2_ Nanosheets Embedded
in a Polymer Ionomer Catalyzing Hydrogen Evolution Reaction. Int. J. Hydrogen Energy.

[23] Zhao L., Song Y., Xie Z., Velez K., Liu Q., An Q. (2025). Atomic-Level Engineering
of Transition Metal Dichalcogenides for
Enhanced Hydrogen Evolution Reaction. Small
Methods.

[24] Kagkoura A., Karamoschos N., Perivoliotis D. K., García A. P., Gracia-Espino E., Tasis D., Tagmatarchis N. (2023). Bifunctional
Nanostructured Palladium/MoS_x_ Electrocatalyst for Cathode
Hydrogen Evolution Reaction PEM Water Electrolysis and Oxygen Reduction
Reaction. Adv. Sustain. Syst..

[25] Feng Y., Xie Y., Yu Y., Chen Y., Liu Q., Bao H., Luo F., Pan S., Yang Z. (2025). Electronic
Metal-Support Interaction
Induces Hydrogen Spillover and Platinum Utilization in Hydrogen Evolution
Reaction. Angew. Chem., Int. Ed..

[26] Yang Z., Zhao H., Dai X., Nie F., Ren Z., Yin X., Gan Y., Wu B., Cao Y., Zhang X. (2021). Phosphorus-Doping
Induced Electronic Modulation of CoS_2_–MoS_2_ Hollow Spheres on MoO_2_ Film-Mo Foil for Synergistically
Boosting Alkaline Hydrogen Evolution Reaction. Int. J. Hydrogen Energy.

[27] Hua W., Sun H.-H., Xu F., Wang J.-G. (2020). A Review and Perspective
on Molybdenum-Based Electrocatalysts for Hydrogen Evolution Reaction. Rare Metals.

[28] Zhao H., Li Z., Deng J., Dai X., Cui M., Nie F., Zhang X., Ren Z., Wang Y., Song W., Liu J. (2020). Amorphous MoS_2_ Nanosheets
on MoO_2_ Films/Mo
Foil as Free-Standing Electrode for Synergetic Electrocatalytic Hydrogen
Evolution Reaction. Int. J. Hydrogen Energy.

[29] Sharma K. H., Hang D.-R., Bolloju S., Lee J.-T., Wu H.-F., Islam S. E., Chou M. M. C., Liang C.-T., Srivastava R. R. (2021). Two-Dimensional
Molybdenum Trioxide Nanoflakes Wrapped with Interlayer-Expanded Molybdenum
Disulfide Nanosheets: Superior Performances in Supercapacitive Energy
Storage and Visible-Light-Driven Photocatalysis. Int. J. Hydrogen Energy.

[30] Thongam D. D., Hang D.-R., Liang C.-T., Huang H.-C., Chou M. M. C. (2025). Ni,
Co, and Mo-Based Trimetallic and Bimetallic Oxide Nanocomposites as
Cost-Effective Bifunctional Electrocatalysts for Coupled Methanol
Oxidation and Hydrogen Evolution. J. Electroanalytic.
Chem..

[31] Islam S. E., Hang D.-R., Liang C.-T., Sharma K. H., Huang H.-C., Chou M. M. C. (2023). Trimetallic Ni–Co–Mo
Nanoparticles Supported
on N-Doped Carbon as a Promising Electrocatalyst for the Methanol-Assisted
Hydrogen Evolution Reaction. ACS Appl. Energy
Mater..

[32] Maarisetty D., Hang D.-R., Chou M. M. C., Parida S. (2022). Tuning the Ni/Co Ratios
and Surface Concentration of Reduced Molybdenum States for Enhanced
Electrocatalytic Performance in Trimetallic Molybdates: OER, HER,
and MOR Activity. ACS Appl. Energy Mater..

[33] Najafi L., Bellani S., Oropesa-Nuñez R., Brescia R., Prato M., Pasquale L., Demirci C., Drago F., Martín-García B., Luxa J., Manna L., Sofer Z., Bonaccorso F. (2020). Microwave-Induced
Structural Engineering
and Pt Trapping in 6R-TaS_2_ for the Hydrogen Evolution Reaction. Small.

[34] Luxa J., Mazánek V., Pumera M., Lazar P., Sedmidubský D., Callisti M., Polcar T., Sofer Z. (2017). 2H→1T Phase
Engineering of Layered Tantalum Disulfides in Electrocatalysis: Oxygen
Reduction Reaction. Chem.—Eur. J..

[35] Beydaghi H., Najafi L., Bellani S., Bagheri A., Martín-García B., Salarizadeh P., Hooshyari K., Naderizadeh S., Serri M., Pasquale L., Wu B., Oropesa-Nuñez R., Sofer Z., Pellegrini V., Bonaccorso F. (2021). Functionalized
Metallic Transition Metal Dichalcogenide (TaS_2_) for Nanocomposite
Membranes in Direct Methanol Fuel Cells. J.
Mater. Chem. A.

[36] Achari A., Bekaert J., Sreepal V., Orekhov A., Kumaravadivel P., Kim M., Gauquelin N., Balakrishna Pillai P., Verbeeck J., Peeters F. M., Geim A. K., Milošević M. V., Nair R. R. (2022). Alternating
Superconducting and Charge Density Wave Monolayers within Bulk 6R-TaS_2_. Nano Lett..

[37] Lacinska E. M., Furman M., Binder J., Lutsyk I., Kowalczyk P. J., Stepniewski R., Wysmolek A. (2022). Raman Optical Activity of 1T-TaS_2_. Nano Lett..

