# Supplementary material for: 6R-TaS2 Anchored on Mo Foil as a Robust Electrocatalyst for Hydrogen Evolution
Source: ACS Appl Mater Interfaces. 2025 Dec 15;17(51):69571–8. doi: 10.1021/acsami.5c22479 (PMC12754742; doi:10.1021/acsami.5c22479)
Supplement: Supplementary file 1 [file am5c22479_si_001.pdf]

Supporting Information

6R-TaS_2 Anchored on Mo Foil as a Robust Electrocatalyst for Hydrogen Evolution

Antonia Kagkoura, Filipa M. Oliveira, Kseniia Mosina, Jan Luxa and Zdeněk Sofer**

Department of Inorganic Chemistry, University of Chemistry and Technology Prague, Technická 5, 166 28 Prague 6, Czech Republic

*Antonia Kagkoura - Email: kagkourn@vscht.cz

*Zdeněk Sofer - Email: zdenek.sofer@vscht.cz

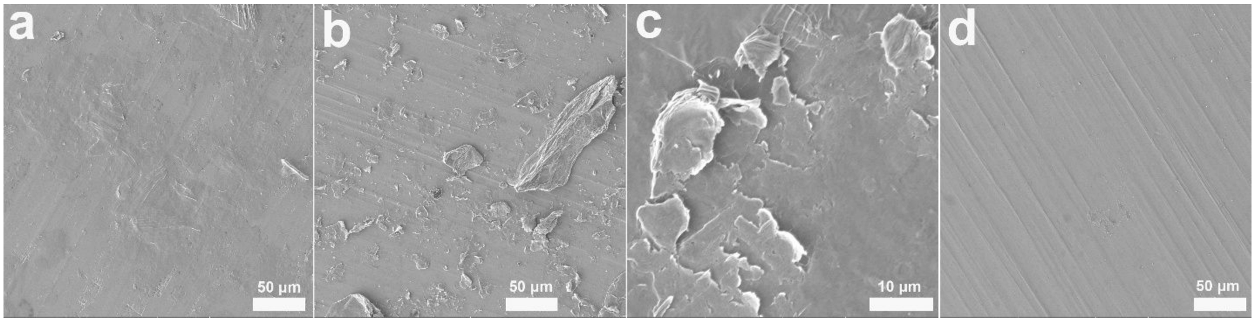

Figure S1. SEM images of (a) 0.4 TaS_2/Mo, (b) 0.8 TaS_2/Mo, (c) 1.5 TaS_2/Mo and (d) plain Mo substrate.

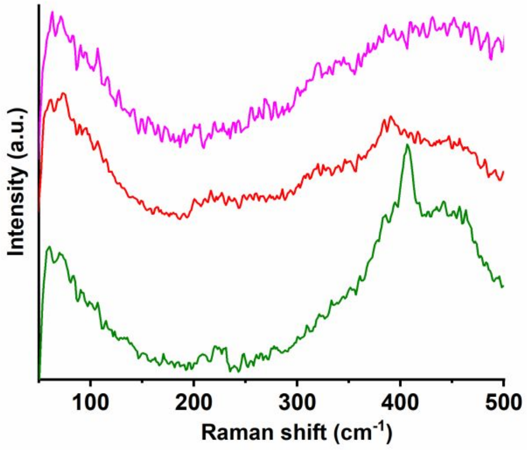

Figure S2. Raman spectra of 0.4 TaS_2/Mo (pink), 0.8 TaS_2/Mo (red) and 1.5 TaS_2/Mo (green).

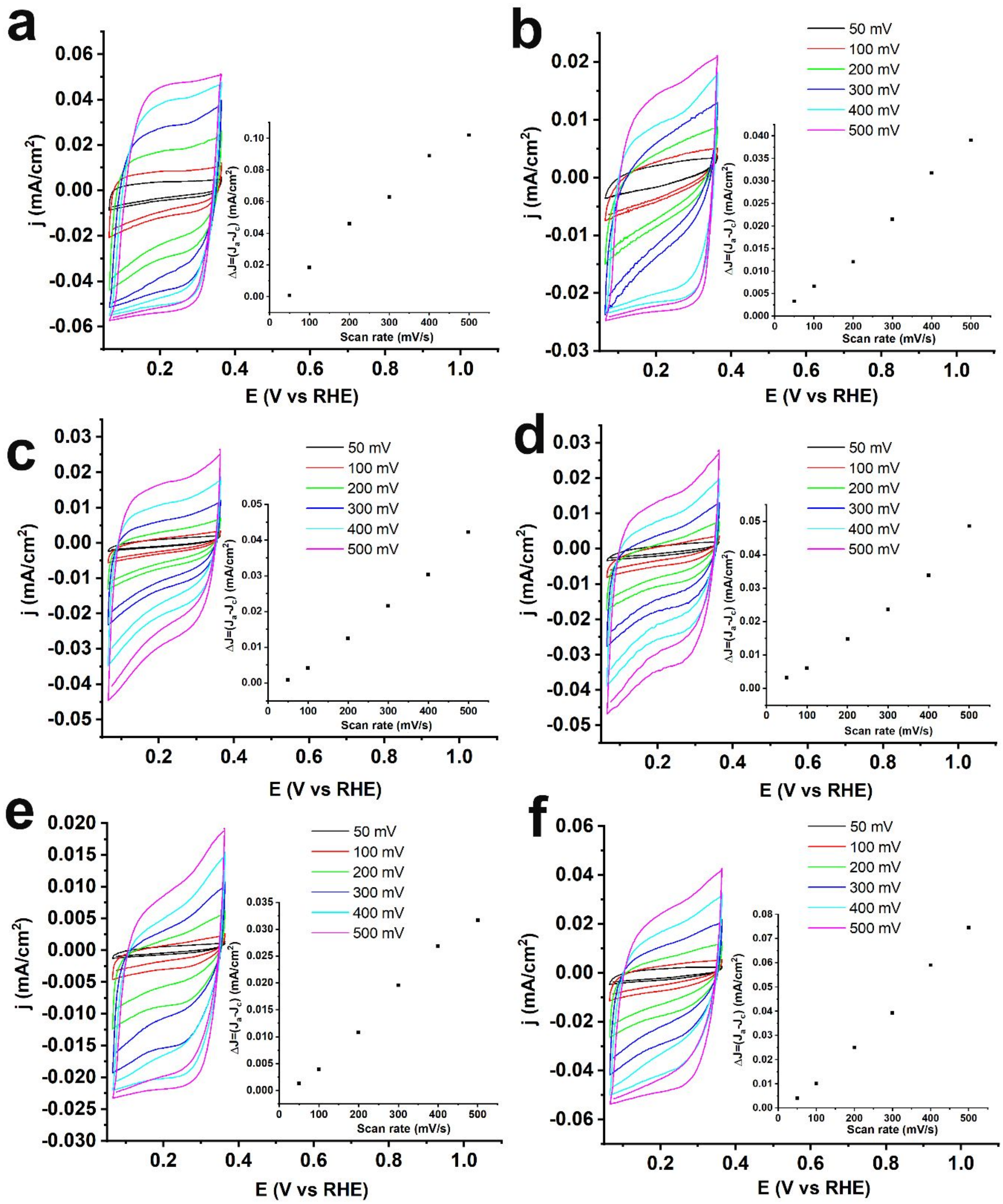

Figure S3. Cyclic voltammograms of (a) 0.8 TaS_2/Mo, (b) 1.5 TaS_2/Mo, (c) 0.4 TaS_2/Mo, (d) annealed Mo (e) Mo and (f) 0.8 TaS_2/Mo after 10,000 cycles in a Ar-saturated aqueous 0.5 M

H_2SO_4 electrolyte, at a rotation speed of 1600 rpm and scan rates from 50 to 500 mV/s. Inset: Scan rate dependence of the current densities for the corresponding materials.

Table S1. Electrocatalytic HER parameters for all tested materials in 0.5 M H_2SO_4.

Material	Onset potential (V vs RHE)	Potential at -10 mA/cm² (V vs RHE)	Tafel slope (mV/dec)	R_{ct} (Ω)	R_s (Ω)	C_{dl} (μF)	ECSA (cm²)	J_{ECSA} (mA/cm^2_{ECSA})**
0.8 TaS_2/Mo	-0.06	-0.15	68	37.5	2.1	1340	5.5	0.45
0.8 TaS_2/Mo*	-0.06	-0.18	148	-	-	-	4.0	0.63
1.5 TaS_2/Mo	-0.10	-0.27	148	76.2	6.9	209	2.04	1.23
0.4 TaS_2/Mo	-0.11	-0.29	160	128	2.0	988	2.28	1.10
Annealed Mo	-0.12	-0.33	206	57.3	3.37	2210	2.48	1.01
Mo	-0.12	-0.34	195	54.6	3.89	3370	1.77	1.41
TaS_2	-0.89	-1.26	252	129.1	8.23	0.491	-	-
Pt/C/Mo	-0.036	-0.08	58	22	2.89	2290	-	-

*after 10,000 cycles

**A_{geo} = 0.25 cm² used; j_{geo} reported at −10 mA·cm⁻²

Table S2. Comparison table of Mo- and TaS_2 based electrocatalysts for HER.

Material	Electrolyte	Overpotential (V vs RHE at $-10\ mA/cm^2$)	Tafel slope (mV/dec)	R_{ct} (Ω)	Ref.
6R-TaS_2/Mo	0.5 M H_2SO_4	150	68	28.7	This work
CoMo	1.0 M KOH	390	43	18.0	1
NiCoMo	1 M KOH	270	87	25.67	1
TaS_2 accordion-like nanosheets	0.5 M H_2SO_4	290	137	85	2
MoS_2/TaS_2/CC	0.5 M H_2SO_4	87	63.2	39	3
$MoSe_2$/TaS_2/CC	0.5 M H_2SO_4	75	54.7	30.4	3
$MoTe_2$/TaS_2/CC	0.5 M H_2SO_4	172	107.1	27.44	3
1T/2H-$MoSe_2$/CFP	0.5 M H_2SO_4	118.75	65.8	24.1	4
TaS_2 NS	0.5 M H_2SO_4	71	40	14	5
$MoTe_2$/$Ti_3C_2T_x$	0.5 M H_2SO_4	293	65	14.7	6
2D-Ta_2Se_2C	0.5 M H_2SO_4	264	91	17.61	7
1T-2H $MoSe_2$/graphene	0.5 M H_2SO_4	153	67	75	8
N–CoMo-M	1 M KOH	112	106.8	-	9
TaS_2/Cu_2S/Cu foil	1 M KOH	144	102	0.3	10

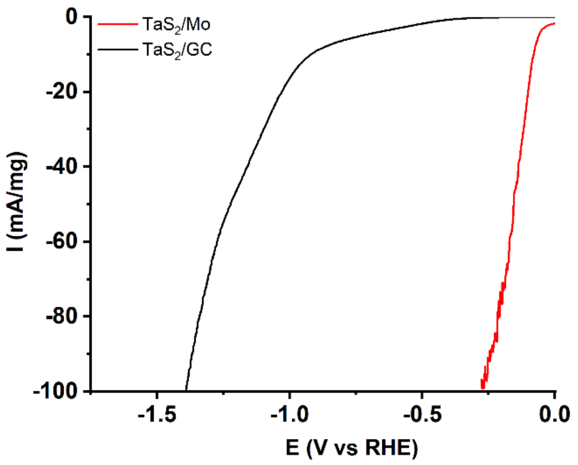

Figure S4. Mass-normalized polarization curves (current density per catalyst mass, mA/mg) for TaS_2/GC (black) and 0.8 TaS_2/Mo electrode (red).

References

(1) Thongam, D. D.; Hang, D.-R.; Liang, C.-T.; Huang, H.-C.; Chou, M. M. C. Ni, Co, and Mo-Based Trimetallic and Bimetallic Oxide Nanocomposites as Cost-Effective Bifunctional Electrocatalysts for Coupled Methanol Oxidation and Hydrogen Evolution. *J. Electroanal. Chem.* **2025**, *996*, 119403. https://doi.org/10.1016/j.jelechem.2025.119403.

(2) Shen, W.; Qiao, L.; Ding, J.; Sui, Y. Constructing 1T–2H TaS_2 Nanosheets with Architecture and Defect Engineering for Enhanced Hydrogen Evolution Reaction. *J. Alloys Compd.* **2023**, *935*, 167877. https://doi.org/10.1016/j.jallcom.2022.167877.

(3) Zhang, Z.; Wang, J.; Li, Y.; Zhang, S.; Xiao, L.; Wang, J.; Qi, J. Hydrothermal Preparation of MoX_2 (X = S, Se, Te)/TaS_2 Hybrid Materials on Carbon Cloth as Efficient Electrocatalyst for Hydrogen Evolution Reaction. *Int. J. Hydrogen Energy* **2023**, *48*, 4207–4219. https://doi.org/10.1016/j.ijhydene.2022.11.002.

(4) Liu, Y.; Liu, S.; Li, H.; Yu, L.; Sun, L.; Xue, J.; Xu, R.; Chen, G. In-Situ Phase Conversion of Composited 1T@2H–$MoSe_2$ Nanosheets with Enhanced HER Performance. *Mater. Chem. Phys.* **2022**, *278*, 125657. https://doi.org/10.1016/j.matchemphys.2021.125657.

(5) Shiraz, H. G.; Vagin, M.; Khan, Z. U.; Chmielowski, R.; Crispin, R.; Berggren, M. TaS_2 Nanosheets Embedded in a Polymer Ionomer Catalyzing Hydrogen Evolution Reaction. *Int. J. Hydrogen Energy* **2025**, *100*, 915–920. https://doi.org/10.1016/j.ijhydene.2024.12.451.

(6) Shinde, P. V.; Mane, P.; Late, D. J.; Chakraborty, B.; Rout, C. S. Promising 2D/2D $MoTe_2/Ti_3C_2T_x$ Hybrid Materials for Boosted Hydrogen Evolution Reaction. *ACS Appl. Energy Mater.* **2021**, *4*, 11886–11897. https://doi.org/10.1021/acsaem.1c02914.

(7) Loni, E.; Majed, A.; Zhang, S.; Thangavelu, H. H. S.; Dun, C.; Tabassum, A.; Eisawi, K.; Urban, J. J.; Persson, P. O. Å.; Montemore, M. M.; Naguib, M. Two-Dimensional Tantalum Carbo-Selenide for Hydrogen Evolution. *ACS Nano* **2025**, *19*, 3185–3196. https://doi.org/10.1021/acsnano.4c09903.

(8) Huang, S.-Y.; Le, P.-A.; Nguyen, V.-T.; Lu, Y.-C.; Sung, C.-W.; Cheng, H.-W.; Hsiao, C.-Y.; Dang, V. D.; Chiu, P.-W.; Wei, K.-H. Surface Plasma–Induced Tunable Nitrogen Doping through Precursors Provides 1T–2H $MoSe_2$/Graphene Sheet Composites as Electrocatalysts for the Hydrogen Evolution Reaction. *Electrochim. Acta* **2022**, *426*, 140767. https://doi.org/10.1016/j.electacta.2022.140767.

(9) Zhao, L.; Wei, L.; He, H.; Zhang, X.; Liu, S.; Wang, J. N-Doped CoMo MOFs-Derived Carbon Nanospheres Electrocatalyst with Co/Mo–N Bonds as Bimetallic Active Sites for Efficient

Hydrogen Evolution Reaction. *Int. J. Hydrogen Energy* **2024**, *62*, 119–126. https://doi.org/10.1016/j.ijhydene.2024.02.361.

(10) Yang, H.; Liu, X.; He, J.; Yan, J.; Bai, Y.; Yin, S.; Li, H.; Yan, H. S-Vacancy-Rich 1T–TaS_2/Cu_2S Heterostructures on Cu Foil for Alkaline Hydrogen Evolution Reaction. *ACS Appl. Nano Mater.* **2025**, *8*, 7243–7255. https://doi.org/10.1021/acsanm.5c00601.
